# Supplementary material for: Intravenous Polyethylene Glycol Alleviates Intestinal Ischemia-Reperfusion Injury in a Rodent Model
Source: Int J Mol Sci. 2023 Jun 28;24(13):10775. doi: 10.3390/ijms241310775 (PMC10341386; doi:10.3390/ijms241310775)
Supplement: Supplementary file 1 [file ijms-24-10775-s001.zip › ijms-2467371-supplementary.pdf]

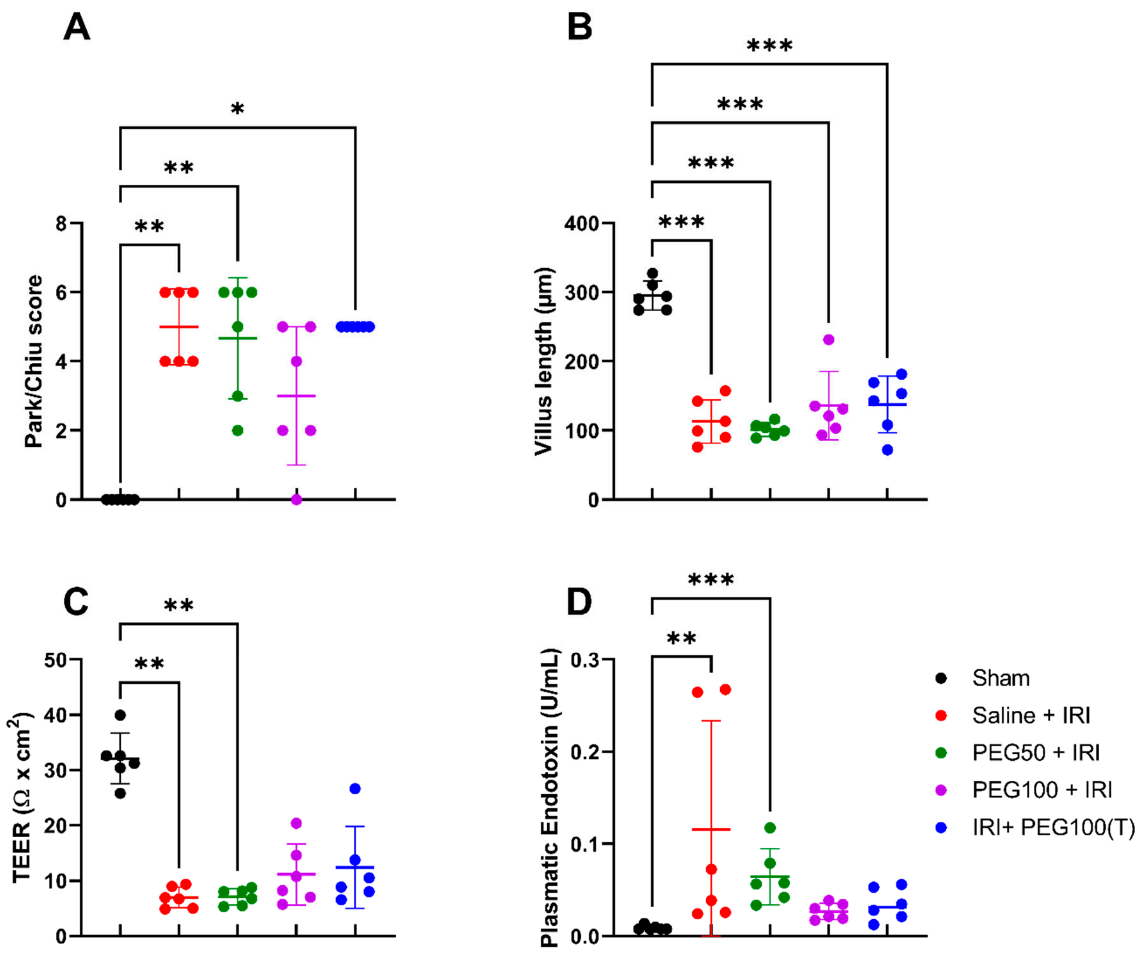

Figure S1 Intestinal epithelial assessment and bacterial translocation

Histopathological effect of IV PEG administration in intestinal IRI was scored according to the Park–Chiu score (**A**) and villus length (**B**). Intestinal epithelial permeability was measured by TEER in an Ussing chamber, which was corrected for villus length (**C**). Bacterial translocation was evaluated by plasmatic endotoxin levels (**D**). (n = 6/group). Statistical analyses were performed by Kruskal-Wallis testing. IRI: Ischemia-Reperfusion Injury; IV: Intravenous; PEG: Polyethylene glycol; TEER: TransEpithelial Electrical Resistance. * $p < 0.05$; ** $p < 0.01$; *** $p < 0.001$.

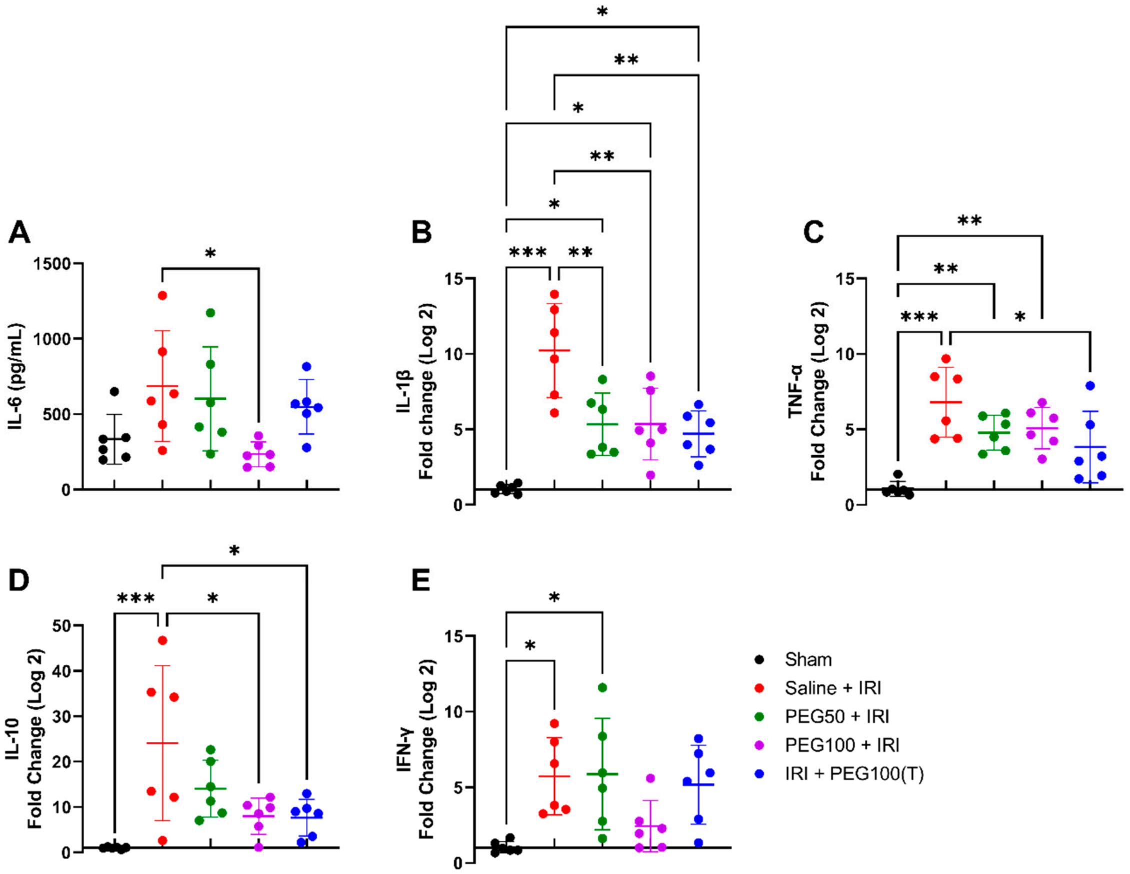

Figure S2: Inflammatory response assessment

Inflammatory modulation by PEG administration was measured by systemic IL-6 (**A**) and intestinal IL-1β (**B**), TNF-α (**C**), IL-10 (**D**), and IFN-γ (**E**). (n = 6/group). Statistical analyses were performed by One-way ANOVA. IFN-γ: Interferon-gamma; IL: Interleukin; IRI: Ischemia-Reperfusion Injury; PEG: Polyethylene glycol; TNF-α: tumor necrosis factor—alfa. * $p < 0.05$; ** $p < 0.01$; *** $p < 0.001$.

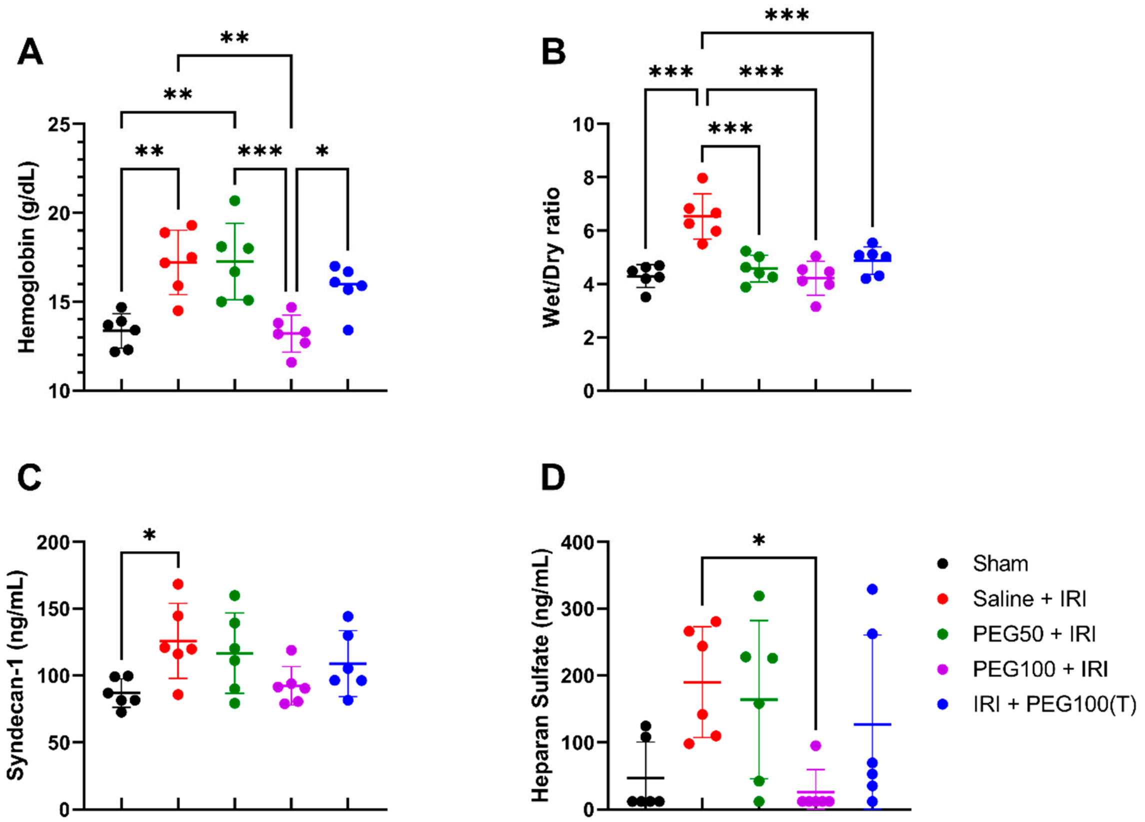

Figure S3 Vascular permeability assessment

Vascular permeability was altered by IV PEG administration, as shown by hemoglobin levels (**A**), reperfusion edema (wet/dry ratio) (**B**), plasmatic endothelial glycocalyx components: syndecan-1 (**C**), and heparan sulfate (**D**). (n = 6/group). Statistical analyses were performed by One-way ANOVA. IRI: Ischemia-Reperfusion Injury; PEG: Polyethylene glycol. * $p < 0.05$; ** $p < 0.01$; *** $p < 0.001$.

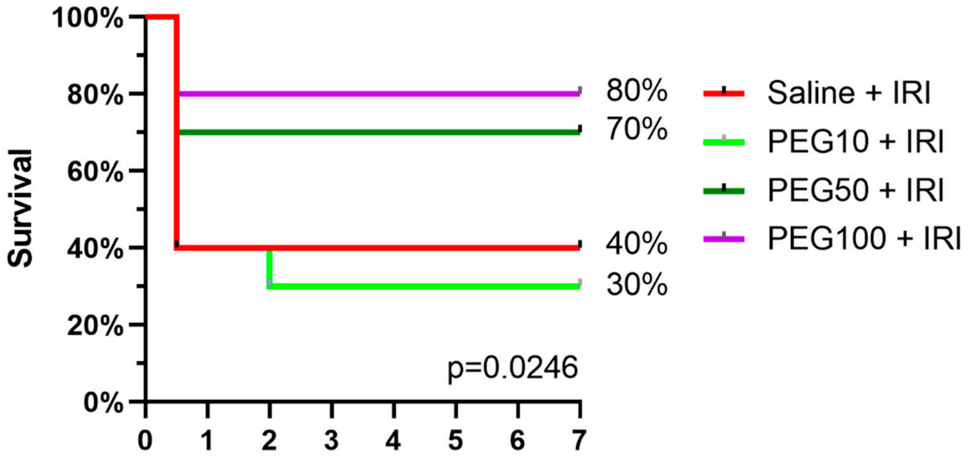

Figure S4: Preliminary dose-dependent survival study

7-days survival, assessed by Kaplan-Meier analysis and Logrank testing for trend, showing a significant trend (p = 0.0246) (n = 10 rats/group). IRI: Ischemia-Reperfusion Injury; PEG: Polyethylene glycol.

Table S1. Preliminary toxicity study: injection in penile vein of male Sprague-Dawley rats

Group	Dosage (mg/kg)	Concentration (% w/v)	Survival (%)	Remark
PEG10	10mg/kg	5g PEG/L (0.5% w/v)	3/3 (100%)	Fluent injection
PEG50	50mg/kg	25g PEG/L (2,5% w/v)	3/3 (100%)	Fluent injection
PEG100	100mg/kg	50g PEG/L (5% w/v)	3/3 (100%)	Fluent injection
PEG200	200mg/kg	100g PEG/L (10% w/v)	0	Too viscous for injection

% w/v: percentage weight/volume; PEG: Polyethylene Glycol
